# Malaria at Parturition in Nigeria: Current Status and Delivery Outcome

**DOI:** 10.1155/2009/473971

**Published:** 2009-07-20

**Authors:** Olugbenga A. Mokuolu, Catherine O. Falade, Adeola A. Orogade, Henrietta U. Okafor, Olanrewaju T. Adedoyin, Tagbo A. Oguonu, Hannah O. Dada-Adegbola, O. A. Oguntayo, Samuel K. Ernest, Davidson H. Hamer, Michael V. Callahan

**Affiliations:** ^1^Department of Pediatrics, University of Ilorin Teaching Hospital, Ilorin 240001, Nigeria; ^2^Department of Clinical Pharmacology, University College Hospital, Ibadan 200001, Nigeria; ^3^Department of Paediatrics, Ahmadu Bello University Teaching Hospital, Kaduna 810003, Nigeria; ^4^Department of Pediatrics, University of Nigeria Teaching Hospital, Enugu 410001, Nigeria; ^5^Department of Medical Microbiology, University College Hospital, Ibadan 200001, Nigeria; ^6^Department of Obstetrics and Gynaecology, Ahmadu Bello University Teaching Hospital, Kaduna 810003, Nigeria; ^7^Center for International Health and Development, Boston University School of Public Health, Crosstown 3rd floor, 801 Massachusetts Avenue, Boston, MA 02118, USA; ^8^Division of Infectious Diseases, Massachusetts General Hospital, 55 Fruit St, Boston, MA 02114, USA

## Abstract

*Background*. To evaluate the current status of malaria at parturition and its impact on delivery outcome in Nigeria. *Methods*. A total of 2500 mother-neonate pairs were enrolled at 4 sites over a 12-month period. Maternal and placental blood smears for malaria parasitaemia and haematocrit were determined. *Results*. Of the 2500 subjects enrolled, 625 were excluded from analysis because of breach in study protocol. The mean age of the remaining 1875 mothers was 29.0 ± 5.1 years. The prevalence of parasitaemia was 17% and 14% in the peripheral blood and placenta of the parturient women, respectively. Peripheral blood parasitaemia was negatively associated with increasing parity (*P* < .0001). Maternal age <20 years was significantly associated with both peripheral blood and placental parasitaemia. After adjusting for covariates only age <20 years was associated with placental parasitaemia. Peripheral blood parasitaemia in the women was associated with anaemia (PCV ≤30%) lower mean hematocrit (*P* < .0001). lower mean birth weight (*P* < .001) and a higher proportion of low birth weight babies (LBW), (*P* = .025). *Conclusion*. In Nigeria, maternal age <20 years was the most important predisposing factor to malaria at parturition. The main impacts on pregnancy outcome were a twofold increase in rate of maternal anaemia and higher prevalence of LBW.

## 1. Introduction

Falciparum malaria in pregnancy is an important cause of maternal and perinatal morbidity and mortality in malaria endemic areas. In pregnancy, malaria is more common, more severe, more atypical, and more fatal [1]. Pregnant women in malarious areas may experience a variety of adverse consequences from malaria infection including anemia and placental accumulation of parasites while their newborns may have low birth weight (LBW) from prematurity and intrauterine growth retardation (IUGR). Other consequences of malaria during pregnancy for the newborn include congenital infection and increased infant mortality (IM) linked either to preterm-LBW or IUGR-LBW [2, 3]. The occurrence of these problems underscores the importance of malaria prevention in pregnancy [1, 4]. Intermittent preventive therapy (IPT) with sulfadoxine-pyrimethamine (SP) has been shown to be superior to chemoprophylaxis or case management in the prevention of malaria in pregnancy [5]. Several other studies have also shown IPT with SP to be highly efficacious and superior to chloroquine prophylaxis [6–8]. The WHO currently recommends a three-prong approach to the control of malaria during pregnancy. These are IPT with SP, ITN and effective case management of clinical infection [9]. 

In Nigeria, several studies focusing on malaria in the peripartum period have been conducted but with small sample sizes and variable findings [10–15]. For instance one study concluded that although malaria parasitaemia is prevalent in our locality, the effects on maternal and fetal wellbeing are comparable in aparasitaemic pregnant women [15] while another study from a similar geographical area in the country concluded that congenital malaria is not uncommon in Lagos nowadays [16]. Mukhtar et al. [16] also reported relatively high rates of maternal, placental and cord blood parasitaemia. Based on these findings the authors recommended that babies born to mothers with malaria should be screened for congenital malaria. The differences in conclusions of these studies may be due to the limited power of the studies owing to their relatively small sample sizes and their restriction to a small section of a large and diverse country such as Nigeria.

With the advent of the Global Fund to fight against Aids, Tuberculosis and Malaria (GFATM), many countries have been able to receive support for scaling up intervention programmes on malaria control including malaria in pregnancy. These activities call for better appraisal of the baseline characteristics of malaria in order to allow for proper impact assessment of the various interventions. Our study was therefore undertaken to evaluate the current status of peripartum malaria and its impact on birth outcomes in Nigeria. This is to serve as a baseline for Nigeria haven only recently developed guidelines for malaria prevention in pregnancy using IPT and ITNs [17].

## 2. Methods

### 2.1. Study Locations

The study was conducted in four geo-political zones of Nigeria, namely North Western (Kaduna), North Central (Ilorin), South Western (Ibadan) and South Eastern zones (Enugu) between April 2003 and March 2004. Malaria is endemic and transmission perennial in all study sites. Detailed description of the study sites have been reported by Falade et al. [18].

### 2.2. Study Population

The subjects of the study were mothers and their newborn babies delivered at the study centres within the study period. In addition to willingness to provide written informed consent, continuous residence within contiguous areas of study the centre for at least 2 years and delivery of a life infant were additional inclusion criteria. 

## 3. Study Design

### 3.1. Sampling Method

This study was prospective and descriptive with enrolment of every consecutive delivery fulfilling the inclusion criteria. However to allow for a representative spread of the subjects across the recruitment period, enrolment was spread out over a twelve calendar month period from April 2003 to March 2004. Monthly recruitment was performed to ensure that mother-baby pairs were enrolled during both high and low malaria transmission season in order to determine the seasonal trends in the prevalence of malaria. Further details of the methodology of this study is in the report by Falade et al. [18].

### 3.2. Procedure

Mother and neonate pairs were enrolled from the labour and delivery unit of each study center. Relevant maternal and neonatal demographic and clinical characteristics were recorded in case record forms. Clinical data obtained include details of labour and delivery, anthropometric parameters of the baby and gestational age. In view of the fact that the subjects were recruited at or near parturition, they could not be placed on Intermittent Preventive Therapy (IPT) using Sulfadoxine-pyrimethamine. Specific note was however made of any malaria preventive measures used by the subjects.

### 3.3. Laboratory Procedure

At delivery, thick and thin blood smears were prepared from maternal finger pricks, placental aspirates (within 1 hour of delivery) and baby heel pricks (within four hours of delivery) after initial cardiopulmonary stabilization. The placental aspirate has been previously reported to have a good correlation with wedge biopsy of the placenta [19]. Blood smears were air dried and subsequently stained with 10% freshly prepared Giemsa stain at Ph 7.2. The stained smears were examined under ×100 oil immersion lens of a light microscope. Malaria diagnosis was based on identification of asexual stages of *Plasmodium* species on the thick blood film while thin smears were used for specie identification. Parasite density was determined by counting the number of parasites against 200 leukocytes (or 500 WBC if no parasite is found) on the thick blood film and converted to parasites/*μ*L using an assumed total white blood cell count of 8000 *μ*L [20]. The packed cell volume (PCV) was determined using blood collected into heparinized capillary tubes and spun with a Hawksley micro-hematocrit centrifuge for 5 minutes.

### 3.4. Quality Assurance

A procedure manual was produced before study initiation to standardize methodology at all study sites. Baseline training for microscopists and investigators was done. A pilot study was also conducted to evaluate the study process and detect potential site specific problems in order to harmonize the study protocol. Prestudy and mid study (within 3 months) quality assurance of the slides were done in a national reference laboratory. Continuous quality control on a monthly basis was also done, by randomly selecting 10 slides for comparison in the institution's main laboratory by a consultant haematologist or the chief technologist.

### 3.5. Ethical Approval

All centres involved in the study have Federal Wide Assurance (FWA) certification. Ethical approval was provided by the Joint University of Ibadan/University College Hospital Ethical Review Board (Ibadan), the University of Ilorin Ethical Review Committee (Ilorin), Ahmadu Bello University Ethical Review Board (Kaduna) and University of Nigeria Teaching Hospital Enugu Institutional Review Committee. In addition, ethics approval was obtained from the Boston University Institutional Review Board. Written informed consent was obtained from each study volunteer or from the study participant's mother or legal guardian (spouse or grandmother) for study volunteers under 18 years of age. Continuous ethical review was carried out for each year the study was active.

### 3.6. Data Collection and Management

Data was collected and recorded into precoded case record forms. The completed case record forms were cross-checked for errors and missing variables by the supervisor. Thereafter, the data was entered into the computer by two data entry clerks using EPI-INFO 6d (Centre for Disease control, Atlanta, USA) programmed with appropriate CHECK commands to minimize error during data entry. 

Relationships between continuous independent variables (e.g., age, anthropometric measurements, temperature, and placental weight) and the outcome variables were tested using the Student's *t*-test or the ANOVA where the assumptions were met. Association between categorical variables was tested using the Chi-square test or Fishers' exact test where applicable. For significant associations, the odds ratio (OR) and the 95% confidence interval (CI) were computed to determine the association between a risk factor and particular categorical outcome variable. For all statistical analysis a *P*-value less than .05 was considered significant.

For the purpose of this study the following definitions were applied.

**Low Birth Weight**

Baby with birth weight less than 2500 g.

**Preterm Baby**

Any baby delivered before 37 weeks of gestation. The last menstrual period (LMP) was used for assessing the gestational ages. However this was validated with either the first trimester ultrasound assessment of the gestational age or the Ballard chart. Any discrepancies in excess of 2 weeks of the gestational age by LMP resulted in the exclusion of the subject.

## 4. Results

### 4.1. Study Population

A total of 2500 subjects were recruited from the four study centres. However due to the problems with buffering conditions for Giemsa stain and resulting deterioration of the blood smears at the Enugu research center, blood smears could not undergo quality assurance procedure. All mother-baby pairs enrolled at that site were excluded from analysis leaving the final sample size analyzed in this report at 1875 mother-baby pairs.

The majority, 1369 (73%), of the women had no previous history of abortion while 18% of the women had experienced at least one episode of abortion prior to the index pregnancy and 2.5% had had between 2-3 previous abortions. The women enrolled in the study were aged between 14–50 years. The distribution of the age, parity and pregnancy outcome is shown in Table 1. Approximately a third of the pregnant women were primiparous. The overall prevalence of LBW babies was 6.8%. There was however a significant reduction in the proportion of LBW babies with increasing parity (*P* = .026). The proportion of preterm deliveries was 10% and it was not associated with parity.

### 4.2. Prevalence and Risk Factors for Maternal Parasitaemia

#### 4.2.1. Prevalence

Of the 1875 women, there were a total of 404 (maternal and placental) positive smears, thus giving a peripartum prevalence of malaria of 21.6%. Those with positive peripheral smears were 319 (17%) while those with positive placental smears were 267 (14.2%). On the basis of the detection of parasites in either peripheral blood or the placenta, 27.2% of primigravidae, 17.9% of secundigravidae, and 19.4% of multigravidae had peripartum malaria parasitaemia. These differences were statistically significant (Table 1).

#### 4.2.2. Maternal Age

**Figure 1:**
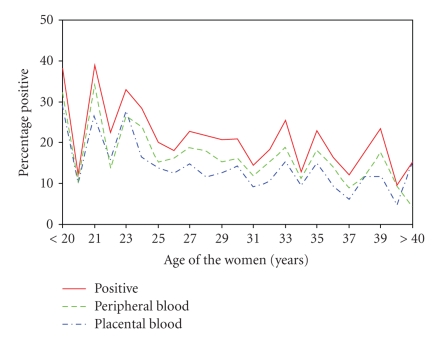
Distribution of peripheral parasitaemia in Nigerian women.

Figure 1 shows the distribution of parasitaemia by maternal age. There was a steady decline in the prevalence of parasitaemia with age from an average of about 35% in those less than 25 years to an average of 15% in women 40 years and older. Maternal age less than 20 years was also significantly associated with both peripheral (*P* = .016; OR = 2.3: 95% CI = 1.2–4.9) and placental parasitaemia (*P* = .01; OR = 2.6: 95% CI = 1.2–5.4).

#### 4.2.3. Parity

There was also a significant decrease in the prevalence of peripheral and placental parasitaemia with increasing parity (*P* = .0001 in both instances). In each case these differences were most significant between primiparous and multiparous women (women in their 2nd pregnancy and above) (Table 2).

#### 4.2.4. Multivariate Analysis

A linear regression of the age of the women and parity against maternal parasitaemia (peripheral and placental) however showed only the age of the women was significantly associated parasitaemia in the women (the contributions were *t* = 3.27; *P* = .001 for age: *t* = −0.75; *P* = .45 for parity).

### 4.3. Effect of Parasitemia on Maternal and Babies Hematocrit

The mean (SD) haematocrit of the women with patent parasitaemia was 34% ± 5.0 while that for those without patent parasitaemia was 37% ± 5.4 (*F*-stat = 46.4, *P* = .0001).

#### 4.3.1. Maternal Anaemia

Maternal parasitaemia was strongly associated with anaemia in the mothers. The presence of patent parasitaemia; peripheral, placental or combined placental/peripheral, had a twofold risk of resulting in significant maternal anaemia (hematocrit ≤30%). When this was stratified by parity there was no significant difference in the mean haematocrit of primigravidae with positive parasitaemia and multigravidae with positive parasitemia (Tables 3  and 4).

#### 4.3.2. Babies' Mean Haematocrit

Peripheral and placental parasitaemia in the women did not affect the mean haematocrit of the babies (Tables 3  and 4). 

### 4.4. Effect of Parasitaemia on Pregnancy Outcome

#### 4.4.1. Birth Weight

There was discordance with respect to peripheral and placental parasitaemia on the babies' birth weight. The observed significant differences were with respect to peripheral parasitaemia. Peripheral parasitaemia was associated with significantly lower mean birth weight 3077 g ± (523) versus 3175 g ± (477) among babies born to mothers with patent peripheral parasitemia and those without; *F*-stat = 10.7, *P* = .001. Low birth weight (LBW) occurred in 128/1875 deliveries thus giving a prevalence of 6.8%. Peripheral parasitaemia was also significantly associated with LBW babies (31/311 [9.97%] versus 97/1556 [6.2%]; *P* = .025, OR = 1.6 [1.1–2.5]).

#### 4.4.2. Other Outcomes

In contrast, preterm deliveries and Apgar scores at 1 and 5 minutes were not associated with peripheral parasitaemia in the mother.

## 5. Discussion

In this study, we found that 21% of mothers had peripheral (maternal) and/or placental parasitaemia at parturition. Previous studies from Nigeria reported parasitaemia rates among pregnant women or at parturition ranging from 24.8–80% [15, 21–25]. Studies from other malaria endemic parts of Africa have also reported large variations in the occurrence of malaria parasitaemia among the pregnant women. For instance, in Cameroon; Walker-Abbey et al. [26] reported a prevalence of 82.4% while Tako et al. [27] reported a total malaria positivity rate of 21.5%. The wide ranges in reported prevalence of malaria may be due to multiple factors. One factor is the method of diagnosis. The studies that reported very high rates, that is, >70%, were those that involved the use of PCR for parasite detection. In one of such studies with 82% prevalence, only 27.5% were detectable by microscopy while parasitaemia was sub-microscopic in over 50% [26]. Other factors that may explain this variation include intensity of transmission, study population characteristics (age, parity, HIV status), use of preventive measures (e.g., IPT, ITNs), and study design. Many studies had small sample sizes and were restricted to just a single site. This was a multicentre study across the major geographic and ecological zones of Nigeria and would therefore be expected to be more representative of the true situation of malaria at parturition in the country. To the best of the authors' knowledge this is the largest study of a multicentre dimension to be conducted on peripartum malaria in Nigeria.

In this study, parasitaemia at the time of delivery was found to be associated with low parity and maternal age. Several other studies have reported similar associations [27–29]. A multivariate analysis however revealed that only the age of the women less than 20 years was significantly associated with parasitaemia in the women. This finding was also described by Tako et al. [27] and Saute et al. [29]. In both of these studies multivariate analysis found that younger maternal age, (teens to less than 25 years), was significantly associated with placental parasitaemia after adjusting for confounding variables like parity. The authors observed that younger but not older first time mothers were more likely to have placental malaria [27] or peripheral blood malaria [29]. In the light of this finding Tako et al. [27] suggested that pregnancy-associated immunity and naturally acquired immunity may differ among women in Yaoundé, Cameroon. The hypothesis was that the development of pregnancy associated immunity, for example, production of antibodies that inhibit adherence of placental parasites to chondroitin sulphate A, may be very important in women <25 years of age who have lower levels of acquired immunity. While older women living in such endemic areas may have obtained adequate immunity following repeated exposures to eliminate any infecting malaria parasite and are thus less dependent on anticytoadherent antibodies [27]. Whereas this hypothesis is plausible, it is not certain whether it will also explain the fact that the observed relationship is not limited to only placental parasitaemia since the same was true for parasitaemia in the maternal peripheral blood. 

Parasitaemia in the mothers was found to be significantly associated with lower maternal hematocrit. This finding is not unexpected as the association of malaria in pregnancy and low hematocrit has been recognized and reported by previous workers [14, 30]. The drop in hematocrit occurs as a result of the fact that parasitized and unparasitized erythrocytes are destroyed by the spleen during malaria infection. It is however known that using drugs that are normally effective against malaria within a locality significantly reduces the occurrence of this anaemia [31].

The main effect of maternal parasitaemia on the babies is the reduction in the birth weight. This is consistent with the observations from other malaria endemic countries [32–35]. Considering that a needle aspiration technique was applied for placenta parasite detection, it was not possible, in this study, to distinguish between acute and chronic stages of the placental infection. The impact of malaria during pregnancy on LBW in sub-Sahara Africa has been extensively reviewed [36, 37]. It has been estimated that in areas where malaria is endemic, about 19% of LBW infants are due to malaria and 6% of infant deaths are due to LBW caused by malaria. These estimates imply that around 100 000 infant deaths each year could be due to LBW caused by malaria during pregnancy in areas of malaria endemicity in Africa [37]. 

The observed impact of malaria on the mother and their newborns add justification for promoting use of malaria preventive measure in pregnancy. The tools for achieving effective malaria control are now available; these include use of ITNs, IPT, and effective treatment. The use of these tools is also being facilitated with the recent production of a number of treatment guidelines and policy documents [17, 38, 39]. In the present study the level of use of malaria preventive measures was generally low. It is essential that all stakeholders combine efforts to ensure successful implementation in the deployment of these various tools in order to achieve a reduction in the burden of malaria in pregnancy in Nigeria.

## 6. Conclusions

In Nigeria, 1 in every 5 women has malaria (maternal and/or placental) parasitaemia at delivery. Maternal age less than 20 years was the most important predisposing factor. Maternal anaemia, reduction in maternal hematocrit, mean birth weight and a higher proportion of LBW babies were the major deleterious outcomes of malaria in the peripartum period. The findings of this large multicentre study which recorded a prevalence of 21.6% for patent parasitaemia (mother and/or placenta) underscores the need for a focused and concerted effort to address the control of malaria during pregnancy in Nigeria.

## Figures and Tables

**Table 1:** Characteristics of the study population, pregnancy outcome and malaria status.

	All women	Number of pregnancies			*P*-value**
		1	2	≥3	
No. of women	1875	604	457	814	
Percentage	100%	32.2%	24.4%	43.4%	

Age in years					
Mean ± SD	29.0 ± 5.1	25.7 ± 4.0	28.0 ± 3.8	32.1 ± 4.6	.0001^+^

*Malaria Status*					
% Peripheral blood	17.0	21.7	14.0	15.3	.001*
% Placental blood	14.2	18.4	11.4	12.8	.002*
Total % malaria positive^*#*^	21.6	27.2	17.9	19.4	<.001*
Mean parasite density (/*μ*L) in peripheral blood ±SD	456 ± 5563	783.8 ± 5873	494.1 ± 8773	191 ± 1552	NS
Mean parasite density (/*μ*L) placenta ±SD	1093 ± 15321	1504 ± 17589	985 ± 18470	847 ± 10950	NS

*Delivery outcome*					
% Low birth weight	6.8	8.9	6.8	5.3	.026*
% Preterm delivery	10.0	9.3	10.1	10.5	.71*

^+^Wilcoxon rank-sum test; *Chi-squared test; ^*#*^Parasites were detected in the peripheral and/or placental blood. **Comparisons were between the three categories of number of pregnancies.

**Table 2:** Risk factors for peripartum parasitaemia among parturient mothers in Nigeria.

Characteristics	Parasitaemia positive	Parasitaemia negative	*n*	*P*	OR (95% CI)
*Peripheral blood*					
Maternal age <20 years	11 (32.4)	23 (67.6)	1875	.016	2.3 (1.2–4.9)
Maternal age ≥20 years	308 (16.7)	1533 (83.3)			

*Placental*					
Maternal age <20 years	10 (29.4)	24 (70.6)	1875	.01	2.6 (1.2–5.4)
Maternal age ≥20 years	257 (14.0)	1584 (86.0)			

*Peripheral blood*					
Primiparous	131 (21.7)	473 (88.3)	1875	.0001	1.5 (1.2–2.0)
Multiparous	188 (14.8)	1083 (85.2)			

*Placental*					
Primiparous	111 (18.4)	493 (81.6)	1875	.0001	1.6 (1.2–2.1)
Multiparous	156 (12.3)	1115 (87.7)			

**Table 3:** Effect of maternal parasitaemia on mean hematocrit and neonatal birth weight.

Parameter	Parasitaemia maternal		*P*-value
Mean (±SD)	Positive	Negative	
Maternal HCT (%)	34.3 (±5.1)	36.5 (±5.4)	.0001
Babies' HCT (%)	52.3 (±7.9)	52.6 (±8.3)	.663
Birth weight (g)	3078 (±523)	3175 (±477)	.001

**Table 4:** Relationship between parasitaemia and anaemia in the mothers.

Maternal parasitaemia	Anaemia	No anaemia	*n*	*P*	OR (95% CI)
*Peripheral and placental blood *					
Positive	47 (25.8)	135 (74.2)	1875	.0001	2.1 (1.4–2.9)
Negative	244 (14.5)	1439 (85.5)			

*Peripheral blood*					
Positive	79 (24.8)	240 (75.2)	1875	.0001	2.1 (1.5–2.8)
Negative	212 (13.7)	1334 (86.3)			

*Placental blood *					
Positive	66 (24.7)	201 (75.3)	1875	.0001	2.0 (1.5–2.7)
Negative	225 (14.1)	1373 (85.9)			

## References

[1] Shulman CE, Dorman EK (2003). Importance and prevention of malaria in pregnancy. *Transactions of the Royal Society of Tropical Medicine and Hygiene*.

[2] Steketee RW, Nahlen BL, Parise ME, Menendez C (2001). The burden of malaria in pregnancy in malaria-endemic areas. *American Journal of Tropical Medicine and Hygiene*.

[3] Das LK (2000). Malaria during pregnancy and its effects on foetus in a tribal area of Koraput District, Orissa. *Indian Journal of Malariology*.

[4] Singh N, Shukla MM, Sharma VP (1999). Epidemiology of malaria in pregnancy in central India. *Bulletin of the World Health Organization*.

[5] Kayentao K, Kodio M, Newman RD (2005). Comparison of intermittent preventive treatment with chemoprophylaxis for the prevention of malaria during pregnancy in Mali. *Journal of Infectious Diseases*.

[6] Rogerson SJ, Chaluluka E, Kanjala M, Mkundika P, Mhango C, Molyneux ME (2000). Intermittent sulfadoxine-pyrimethamine in pregnancy: effectiveness against malaria morbidity in Blantyre, Malawi, in 1997–99. *Transactions of the Royal Society of Tropical Medicine and Hygiene*.

[7] van Eijk AM, Ayisi JG, ter Kuile FO (2004). Effectiveness of intermittent preventive treatment with sulphadoxine-pyrimethamine for control of malaria in pregnancy in western Kenya: a hospital-based study. *Tropical Medicine and International Health*.

[8] Parise ME, Ayisi JG, Nahlen BL (1998). Efficacy of sulfadoxine-pyrimethamine for prevention of placental malaria in an area of Kenya with a high prevalence of malaria and human immunodeficiency virus infection. *American Journal of Tropical Medicine and Hygiene*.

[9] WHO A strategic frame work for malaria prevention and control during pregnancy in the African sub-region.

[10] Ibhanesebhor SE, Okolo AA (1992). Placental malaria and pregnancy outcome. *International Journal of Gynecology and Obstetrics*.

[11] Fleming AF (1990). Antimalarial prophylaxis in pregnant Nigerian women. *The Lancet*.

[12] Egwunyenga OA, Ajayi JA, Duhlinska-Popova DD (1995). Transplacental passage of *Plasmodium falciparum* and seroevaluation of newborns in northern Nigeria. *The Journal of Communicable Diseases*.

[13] Egwunyenga OA, Ajayi JA, Popova-Duhlinska DD, Nmorsi OPG (1996). Malaria infection of the cord and birthweights in Nigerians. *Central African Journal of Medicine*.

[14] Egwunyenga OA, Ajayi JA, Duhlinska-Popova DD (1997). Malaria in pregnancy in Nigerians: seasonality and relationship to splenomegaly and anaemia. *Indian Journal of Malariology*.

[15] Sule-Odu AO, Ogunledun A, Olatunji AO (2002). Impact of asymptomatic maternal malaria parasitaemia at parturition on perinatal outcome. *Journal of Obstetrics and Gynaecology*.

[16] Mukhtar MY, Lesi FEA, Iroha EU, Egri-Okwaji MTC, Mafe AG (2006). Congenital malaria among inborn babies at a tertiary centre in Lagos, Nigeria. *Journal of Tropical Pediatrics*.

[17] Federal Ministry of Health (2005). National Guidelines and Strategy for malaria prevention and control during in pregnancy.

[18] Falade CO, Mokuolu OA, Okafor HU (2007). Epidemiology of congenital malaria in Nigeria: a multi-centre study. *Tropical Medicine and International Health*.

[19] Sowunmi A, Abohweyere AEJ, Akindele JA, Ilesanmi AO, Falade CO, Oduola AMJ (1996). Comparison of the incision and aspiration methods for the diagnosis of placental malaria infection. *Journal of Obstetrics and Gynaecology*.

[20] Trape J-F, Rogier C (1996). Combating malaria morbidity and mortality by reducing transmission. *Parasitology Today*.

[21] Egwunyenga OA, Ajayi JA, Duhlinska-Popova DD (1997). Transplacental passage of *Plasmodium falciparum* and seroevaluation of newborns in northern Nigeria. *Southeast Asian Journal of Tropical Medicine and Public Health*.

[22] Anorlu RI, Odum CU, Essien EE (2001). Asymptomatic malaria parasitaemia in pregnant women at booking in a primary health care facility in a periurban community in Lagos, Nigeria. *African Journal of Medicine and Medical Sciences*.

[23] Onyenekwe CC, Meludu SC, Dioka CE, Salimonu LS (2002). Prevalence of asymptomatic malaria parasitaemia amongst pregnant women. *Indian Journal of Malariology*.

[24] Okwa OO (2003). The status of malaria among pregnant women: a study in Lagos, Nigeria. *African Journal of Reproductive Health*.

[25] Obiajunwa PO, Owa JA, Adeodu OO (2005). Prevalence of congenital malaria in Ile-Ife, Nigeria. *Journal of Tropical Pediatrics*.

[26] Walker-Abbey A, Djokam RRT, Eno A (2005). Malaria in pregnant Cameroonian women: the effect of age and gravidity on submicroscopic and mixed-species infections and multiple parasite genotypes. *American Journal of Tropical Medicine and Hygiene*.

[27] Tako EA, Zhou A, Lohoue J, Leke R, Taylor DW, Leke RFG (2005). Risk factors for placental malaria and its effect on pregnancy outcome in Yaounde, Cameroon. *American Journal of Tropical Medicine and Hygiene*.

[28] Bouyou-Akotet MK, Ionete-Collard DE, Mabika-Manfoumbi M (2003). Prevalence of *Plasmodium falciparum* infection in pregnant women in Gabon. *Malaria Journal*.

[29] Saute F, Menendez C, Mayor A (2002). Malaria in pregnancy in rural Mozambique: the role of parity, submicroscopic and multiple *Plasmodium falciparum* infections. *Tropical Medicine and International Health*.

[30] Shulman CE, Marshall T, Dorman EK (2001). Malaria in pregnancy: adverse effects on haemoglobin levels and birthweight in primigravidae and multigravidae. *Tropical Medicine and International Health*.

[31] Garner P, Gulmezoglu AM (2000). Prevention versus treatment for malaria in pregnant women. *Cochrane Database of Systematic Reviews*.

[32] Wort UU, Warsame M, Brabin B (2006). Birth outcomes in adolescent pregnancy in an area with intense malaria transmission in Tanzania. *Acta Obstetricia et Gynecologica Scandinavica*.

[33] Sarr D, Marrama L, Gaye A (2006). High prevalence of placental malaria and low birth weight in sahelian periurban area. *American Journal of Tropical Medicine and Hygiene*.

[34] N'Dao CT, N'Diaye J-L, Gaye A, Le Hesran J-Y (2006). Placental malaria and pregnancy outcome in a peri urban area in Senegal. *Revue d'Epidemiologie et de Sante Publique*.

[35] Duffy PE, Fried M (2005). Malaria in the pregnant woman. *Current Topics in Microbiology and Immunology*.

[36] Lawn JE, Cousens S, Zupan J (2005). 4 million neonatal deaths: when? Where? Why?. *The Lancet*.

[37] Guyatt HL, Snow RW (2004). Impact of malaria during pregnancy on low birth weight in sub-Saharan Africa. *Clinical Microbiology Reviews*.

[38] Federal Ministry of Health

[39] Federal Ministry of Health

